# Effects of exercise on cardiorespiratory fitness in individuals with MASLD: a systematic review and dose-response meta-analysis

**DOI:** 10.3389/fspor.2026.1854220

**Published:** 2026-06-19

**Authors:** Wei Huang, Yifei He, Hainan Lu, Junyu Wang, Yixin Liang, Lizhaoxi Zeng, Lulu Li, Hongyu Lu, Zhihang Li, Ziyu Wang, Yijun Xie, Peng Duan, Jin Lu

**Affiliations:** 1 Shanghai Customs University, Department of General Education, Shanghai, China; 2 Shanghai Changhai Hospital, Department of Endocrinology, Shanghai, China; 3 Shanghai Changhai Hospital, Department of Nutrition, Shanghai, China; 4 School of Exercise and Health, Shanghai University of Sport, Shanghai, China

**Keywords:** aerobic exercise, cardiorespiratory fitness, dose-response meta-analysis, MASLD, supervised exercise

## Abstract

**Background:**

Cardiorespiratory fitness (CRF), primarily assessed via maximal oxygen uptake (VO₂max) or peak oxygen uptake (VO₂peak) during incremental exercise testing, is a robust predictor of cardiovascular mortality in individuals with metabolic dysfunction-associated steatotic liver disease (MASLD). However, the exercise volume associated with greater CRF gains in this population remains poorly defined. Therefore, this study aimed to evaluate the efficacy of supervised structured exercise on CRF in MASLD individuals and characterize the dose-response relationship using restricted cubic splines.

**Methods:**

We systematically searched PubMed, Embase, and Web of Science through July 18, 2025, for randomized controlled trials comparing supervised exercise with usual care in adults with MASLD. The primary outcome was CRF, measured as VO_2_peak/VO_2_max. Standardized mean differences (SMDs) were pooled using random-effects models. Dose-response analysis was conducted via a Bayesian framework using restricted cubic spline model to identify optimal weekly and cumulative exercise volumes.

**Results:**

Thirteen RCTs comprising 15 exercise-control comparisons involving 356 participants were included. Exercise significantly improved CRF compared with control conditions (SMD = 0.82, 95% CI: 0.54–1.10; I^2^ = 30.1%). In the MD-based analysis restricted to studies reporting CRF in mL/kg/min, exercise increased CRF by 3.52 mL/kg/min (95% CI: 2.04–5.01; I^2^ = 65.5%). Exploratory dose-response analyses suggested non-linear associations, with estimated peak points of approximately 590 MET-min/week for weekly exercise volume and 10,715 MET-min for total cumulative exercise volume.

**Conclusion:**

Exercise interventions improve CRF in individuals with MASLD. Exploratory dose-response analyses suggest that CRF gains may follow a non-linear pattern with increasing exercise volume, but the estimated peak points should be interpreted as model-based indicators rather than definitive prescription thresholds.

**Systematic Review Registration:**

https://www.crd.york.ac.uk/PROSPERO/view/CRD420251243283, PROSPERO CRD420251243283.

## Background

Metabolic dysfunction-associated steatotic liver disease (MASLD), formerly known as non-alcoholic fatty liver disease (NAFLD) (1), has emerged as the most prevalent chronic liver disease globally (2), paralleling the rising epidemics of obesity and type 2 diabetes (3, 4). While liver-related morbidity is a significant concern, cardiovascular disease (CVD) remains one of the leading cause of mortality in patients with MASLD (5). Therefore, management strategies that simultaneously address liver health and mitigate cardiovascular risk are of paramount importance for this population.

Cardiorespiratory fitness (CRF), commonly assessed by maximal oxygen uptake (VO₂max) or peak oxygen uptake (VO₂peak) during incremental exercise testing (6), reflects the body's capacity to transport and utilize oxygen during sustained physical activity and serves as a powerful independent predictor of all-cause and cardiovascular mortality (7). In the context of MASLD, low CRF is strongly associated with an increased risk and prevalence of hepatic steatosis (8). Furthermore, histological evidence indicates that reduced CRF is primarily linked to active necroinflammatory processes and the presence of metabolic dysfunction-associated steatohepatitis (MASH) (9). Pathophysiologically, low CRF in MASLD is likely multifactorial. Evidence from patients with NAFLD/MASLD suggests that VO₂peak is independently associated with hepatic mitochondrial function, adiposity, type 2 diabetes, and cardiovascular factors (10). Consequently, improving CRF is increasingly recognized as a critical therapeutic target to prevent CVD progression and improve long-term prognosis.

Exercise is established as a cornerstone of first-line therapy for MASLD. While numerous studies have confirmed the efficacy of exercise in reducing hepatic steatosis and improving liver enzymes (11, 12), the specific impact of varying exercise characteristics on CRF within the MASLD population remains less defined. Current physical activity guidelines generally recommend 150 min of moderate-intensity activity per week (13), and Keating et al. concluded that a minimum volume of 135 min per week is required to effectively reduce hepatic steatosis (1). However, it remains unclear whether this generic recommendation provides sufficient exercise volume for maximizing cardiorespiratory benefits in individuals with MASLD, and whether such a dose leads to over-training or insufficient stimulation in individuals with MASLD remains to be determined. Crucially, previous meta-analyses on exercise interventions for MASLD have largely treated exercise as a binary variable or focused exclusively on linear associations, thereby failing to capture potential non-linear dose-response relationships (11, 14). Understanding the exact relationship between exercise volume (intensity×duration) and CRF improvement is essential for developing targeted and personalized exercise prescriptions.

Therefore, the objective of this systematic review and meta-analysis was to evaluate the effects of supervised structured exercise interventions on CRF in individuals with MASLD. A key novelty of this study is the application of a dose-response meta-analysis using restricted cubic splines to characterize the dose-response relationship between exercise volume (weekly and total cumulative dose) and CRF. We aimed to evaluate the efficacy of exercise in improving CRF and identify potential dose ranges associated with greater CRF improvement, ultimately providing robust, evidence-based guidance for clinical practice.

## Method

### Protocol and registration

This study adhered to the guidelines of the Preferred Reporting Items for Systematic Reviews and Meta-Analyses (PRISMA) and was registered in the International Prospective Register of Systematic Reviews (CRD420251243283).

### Search strategy

A systematic search was conducted in electronic databases, including PubMed, Web of Science, and Embase, using search terms related to exercise, cardiorespiratory fitness, and MASLD, as detailed in the Supplementary Materials. Studies using the previous NAFLD/NASH terminology were considered eligible when the original study population was clinically consistent with the MASLD framework. The search was restricted to studies available up to July 18, 2025. To minimize the risk of missing relevant studies, the reference lists of all included articles and the bibliographies of systematic reviews published in the past five years were thoroughly examined. All retrieved records were imported into EndNote X9 for management. Titles, abstracts, and full texts were independently screened by two researchers, with any disagreements resolved through discussion or adjudication by a third author.

### Study selection

The study selection criteria were formulated based on the PICOS framework. Studies were eligible for inclusion if they met the following conditions: (1) adult participants of any sex; (2) receipt of supervised structured exercise interventions, including resistance training, aerobic exercise, high-intensity interval training, or combined exercise; (3) a control group receiving usual care or no intervention; (4) outcomes including laboratory-measured cardiorespiratory fitness; (5) randomized controlled trial design; and (6) for studies with multiple intervention arms, inclusion was permitted as long as there was a single exercise-only intervention and a corresponding control group. Studies were excluded if they met any of the following criteria: (1) non-adults (age < 18 years) or patients with other chronic liver diseases (e.g., viral hepatitis or alcoholic liver disease); (2) unstructured or non-supervised physical activity, or combined interventions where the independent effect of exercise could not be isolated; (3) lack of standard measured CRF(VO_2_max or VO_2_peak)or missing key exercise parameters (intensity, duration, frequency) required for MET-min calculation; (4) insufficient data for pooling (e.g., no post-intervention measurements) despite multiple attempts to contact authors; (5) non-randomized trials, reviews, abstracts, or animal studies.

### Data extraction

Data extraction was independently performed by two researchers. The following study characteristics were collected: author, country, year of publication, study design, study groups, sample size (randomized and analyzed), participant characteristics (disease type, age, sex distribution, BMI), intervention details (frequency, intensity, type, duration, and period), and control group information.

Mean scores and standard deviations (SDs) of CRF were extracted to calculate effect sizes. When studies reported standard errors (SEs), SDs were calculated using the formula: SD = SE × √n. If necessary, SDs were estimated from confidence intervals (CIs), t-values, or *p*-values following the Cochrane Handbook guidelines. For studies with incomplete data, the corresponding authors were contacted up to four times within six weeks to retrieve the missing information.

### Calculation

To ensure comparability across studies with varying intensity metrics, exercise intensity was converted into metabolic equivalent values (METs) primarily according to ACSM relative-intensity classifications and the reported exercise prescription parameters, including %VO2peak, %VO2max, %HRmax, %HRR, RPE, exercise mode, session duration, weekly frequency, and intervention duration. The Compendium of Physical Activities was used as a supplementary reference to identify the closest activity category for specific exercise modes. When an intensity range was reported, the midpoint of the range was used for dose calculation. For continuous aerobic exercise, MET values were assigned according to the reported exercise mode and intensity. For interval training, high- and low-intensity segments were calculated separately when segment-specific duration and intensity were available. When such information was unavailable, the reported average session intensity was used. For resistance training, MET values were assigned according to the Compendium category most consistent with the reported training mode and intensity.

Exercise volume was calculated as weekly exercise volume and total cumulative exercise volume. Weekly exercise volume was calculated as MET intensity×weekly exercise duration and expressed as MET-min/week. Total cumulative exercise volume was calculated as weekly volume×intervention duration in weeks and expressed as MET-min. When study duration was reported in months, a standardized conversion of 4 weeks per month was applied. Because MET-based estimates may not fully capture the external load, rest intervals, and neuromuscular demands of resistance training, sensitivity analyses excluding the resistance-training study were conducted in both the conventional meta-analysis and dose-response analysis. All conversions and exercise-dose calculations were independently checked by two reviewers with exercise science expertise, and discrepancies were resolved by consensus.

### Risk of bias assessment

In this study, the quality of included randomized controlled trials was assessed using the Cochrane Risk of Bias Tool, version 2.0 (RoB 2.0). This tool systematically evaluates the risk of bias across five distinct domains: bias arising from the randomization process, bias due to deviations from intended interventions, bias due to missing outcome data, bias in measurement of the outcome, and bias in selection of the reported result. For each domain, we answered a series of specific “signalling questions” and, based on these responses, determined the risk of bias for that domain as “low,” “some concerns,” or “high.” The overall risk of bias for each study was then classified as “low risk,” “some concerns,” or “high risk.” Two independent reviewers performed the assessment, and any discrepancies were resolved by consensus or by a third reviewer.

### Data synthesis

Data synthesis was conducted using R (version 4.5.1). Pooled effects were expressed as standardized mean differences (SMDs) with 95% confidence intervals (CIs). The SMD was selected as the primary effect size because CRF assessment protocols differed across studies, including treadmill-based tests, cycle ergometer-based tests, and laboratory-based submaximal extrapolation, which may influence absolute VO₂peak values. To improve clinical interpretability, pooled results calculated as weighted mean differences (WMDs) are also presented in the main manuscript after excluding Mucinski et al. (2024) (15), which reported CRF in L/min rather than mL/kg/min. We applied a random-effects model using the inverse variance method to account for potential heterogeneity across trials. Between-study heterogeneity was assessed using the I^2^ statistic, with thresholds of 25%, 50%, and 75% corresponding to low, moderate, and high heterogeneity, respectively. For multi-arm trials sharing a common control group, the control sample size was split across comparisons, while the control mean and standard deviation were kept unchanged. For Keating et al. (2015), a sensitivity analysis was conducted by combining the three exercise arms and comparing them with the full control group. Prespecified endpoints were prioritized when multiple time points were reported. For multiple publications from the same registered trial or potentially overlapping study population, only the report with the larger relevant sample size or more complete CRF data was included. Potential small-study effects and publication bias were assessed using Egger's regression test for funnel plot asymmetry. Funnel plots and contour-enhanced funnel plots were visually inspected, and the trim-and-fill method was performed as a sensitivity analysis when asymmetry was observed.

Subgroup analyses were conducted to explore potential moderators of intervention effectiveness. Subgroup cut-offs were selected based on clinical relevance, common exercise-prescription practice, recovery considerations, and the distribution of included comparisons. Specifically, analyses were stratified by intervention duration (>12 weeks vs. ≤12 weeks), exercise frequency (>3 vs. ≤3 sessions/week), intensity (3.0–5.9 METs vs. ≥6.0 METs), exercise type, session duration (<40 vs. ≥40 min), and baseline BMI (<30, 30–35, and >35 kg/m^2^). The 12-week threshold was selected because 12 weeks is a commonly used duration in exercise intervention studies and provided a relatively balanced subgroup distribution. The 3 sessions/week cut-off was selected because three weekly sessions represent a common structured exercise prescription that generally allows alternate-day training and adequate recovery, whereas frequencies above 3 sessions/week reflect higher training density and may involve shorter recovery intervals or consecutive-day training. Session duration, weekly and total cumulative exercise volumes were dichotomized using the median values of the included comparisons as cut-off points.

Following the conventional meta-analysis, dose–response analyses were conducted for CRF, which showed a statistically significant pooled effect. The relationship between exercise dose and CRF improvement was characterized using a Bayesian model-based meta-analysis framework implemented via the MBNMAdose R package, with restricted cubic splines used to flexibly model potential non-linear associations. Exercise dose was operationalized as weekly exercise volume (MET-min/week) and total cumulative exercise volume (MET-min). The primary dose-response analyses were performed using SMDs, and corresponding MD-based analyses were conducted after excluding Mucinski et al. (2024), which reported CRF in L/min rather than mL/kg/min. In addition, the SMD-based RCS analysis was repeated after excluding Keating et al. (2017), the only resistance-training study, to evaluate effect of aerobic exercise on CRF. All procedures adhered to the PRISMA 2020 guidelines and the Cochrane Handbook for Systematic Reviews of Interventions.

## Results

### Characteristics of included studies

**Figure 1:**
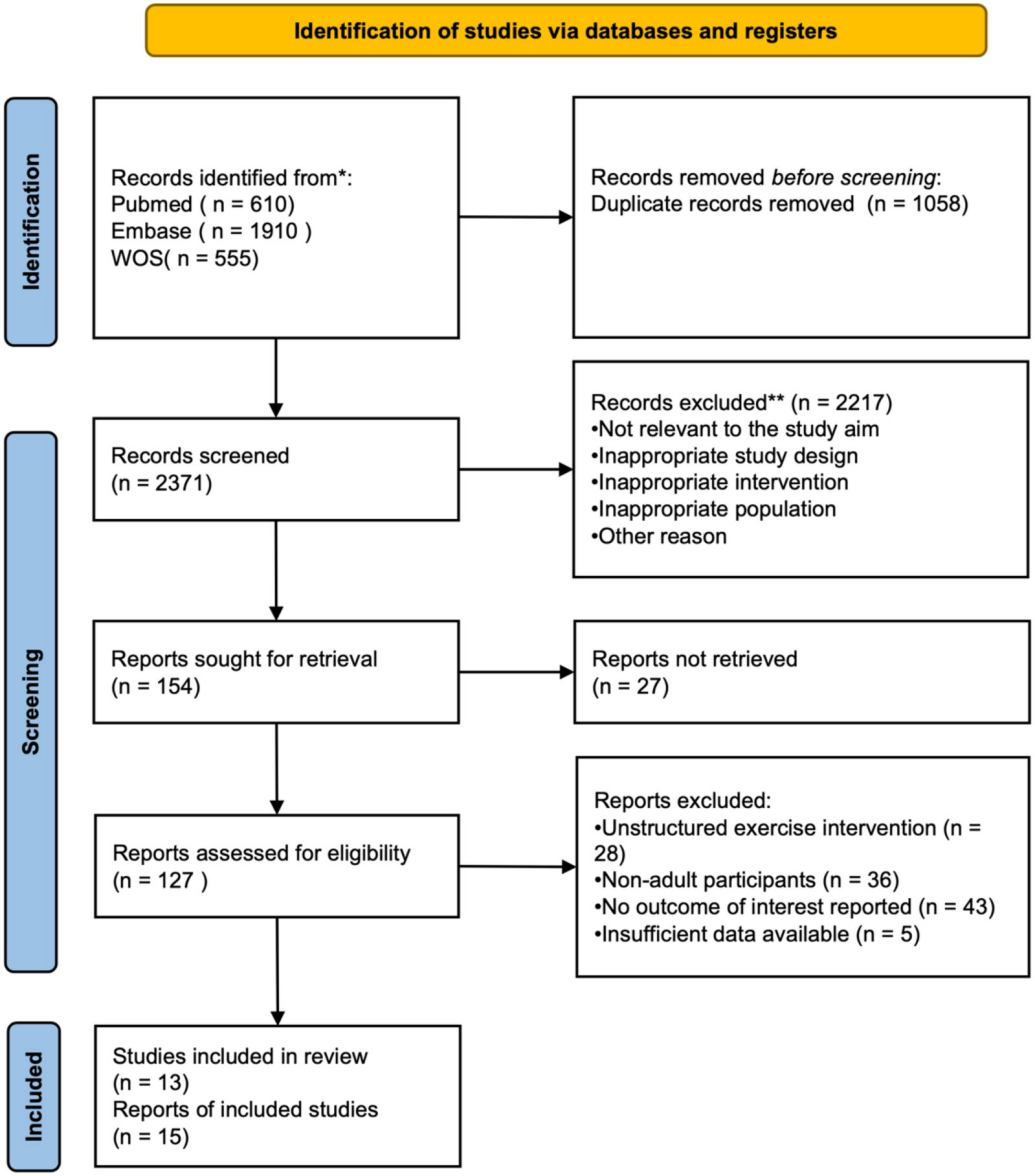
PRISMA flow diagram of the study selection process.

A total of 13 randomized controlled trials comprising 15 exercise-control comparisons were included in the analysis. Figure 1 presents the PRISMA flow diagram depicting the systematic search and study selection process. The initial electronic search yielded 3075 potential articles. After screening the title and abstract, 127 studies were considered eligible for full-text assessment. Ultimately, 13 randomized controlled trials met the inclusion criteria and were included in the meta-analysis. To avoid double-counting, when multiple reports originated from the same trial or were linked to the same registered trial, only one report was retained. Specifically, Stine et al. (2022) was retained instead of Harris et al., and Cuthbertson et al. (2016) was retained instead of Shojaee-Moradie et al. (2016), because these reports provided the larger relevant sample size or more complete CRF data.

The included RCTs, published between 2009 and 2024, enrolled a total of 356 participants, including 211 participants in exercise groups and 145 participants in control groups, with ages ranging from 44.2 to 61.0 years (mean=50). Intervention duration varied from 4 to 40 weeks, with exercise frequencies of 2–5 sessions per week and session lengths ranging from 25.0 to 48.0 min. The intervention duration varied from 4 to 40 weeks.

Among the included comparisons, 11 involved continuous aerobic training, 3 involved high-intensity interval training (HIIT), and 1 involved resistance training. Details were presented in Table 1.

**Table 1:** Characteristics of included studies.

First author and country	Subjects	Demographics	Exercise frequency	Exercise intensity	Exercise type	Time (min)	Duration (weeks)	Baseline CRF	CRF assessment method
Johnson et al. 2009 (24) AUS	Obese subjects *N* = 23 M/F: 15/8	Age CON: 47.3 yrs Exe: 49.1 yrs BMI CON:31.1 kg/m^2^ Exe: 32.2 kg/m^2^	3	50–70% VO_2_peak	AT	30–45	4	Exe:25.9 mL/kg/min CON:25.0 mL/kg/min	Exercise method: Cycle ergometer Protocol：Physical Work Capacity 170 (PWC−170) sub-maximal test—three 5-min stages at 110–120, 130–140, 150–160 bpm; VO₂peak extrapolated from power–HR relationship of final 60 s at each stage.
Sullivan et al. 2012 (32) USA	Obese subjects *N* = 18 M/F: 5/13	Age CON: 47.5 yrs Exe: 48.6 yrs BMI CON:40.0 kg/m^2^ Exe: 37.1 kg/m^2^	5	45–55%HRR	AT	30–60	16	Exe: 22.8 mL/kg/min CON: 18.5 mL/kg/min	Exercise method: Treadmill Protocol：Modified Balke protocol (constant 3.0 mph; grade +2.5% every 2 min to exhaustion)
Pugh et al. 2013 (28) UK	Adults with MASLD *N* = 11 M/F: NR	Age CON: 51.0 yrs Exe: 45.0 yrs BMI CON:30.0 kg/m^2^ Exe: 31.0 kg/m^2^	4	30–60%HRR	AT	30–45	16	Exe: 26.8 mL/kg/min CON: 22.4 mL/kg/min	Exercise method: Treadmill Protocol：Bruce protocol (2-min warm-up, then 3-min stages with simultaneous speed & grade increments to exhaustion)
Pugh et al. 2014 (29) UK	Sedentary and obese adults *N* = 21 M/F: 11/10	Age CON: 47.0 yrs Exe: 50.0 yrs BMI CON:30.0 kg/m^2^ Exe: 30.0 kg/m^2^	4	30–60%HRR	AT	30–45	16	Exe: 26.7 mL/kg/min CON: 27 mL/kg/min	Exercise method: Treadmill Protocol：Incremental ramp (start 2.2 km/h 0%; speed & grade increased every minute to exhaustion)
Keating et al. 2015 (25) AUS	Sedentary overweight or obese adults *N* = 48 M/F: 17/31	Age CON: 49.1 yrs Exe1: 44.2 yrs Exe2: 45.5 yrs Exe3: 45.6 yrs BMI CON:32.2 kg/m^2^ Exe1:36.3 kg/m^2^ Exe2:33.9 kg/m^2^ Exe3:31.3 kg/m^2^	3	60–70% VO_2_max 50% VO_2_max 50% VO_2_max	AT	30–45 45–60 30–45	8	Exe 1: 21.9 mL/kg/min Exe 2: 24.9 mL/kg/min Exe 3: 22.4 mL/kg/min CON: 21.7 mL/kg/min	Exercise method: Cycle ergometer Protocol：Ramp-incremental test (initial power: 35 W for women, 65 W for men; +25 W every 150 s until volitional exhaustion); VO₂peak calculated from the highest 15-s average in the final minute
Rezende et al. 2016 (30) BRZ	Menopause women with NALFD *N* = 40 M/F: 0/40	Age CON: 54.5yrs Exe: 56.2 yrs BMI CON:32.0 kg/m^2^ Exe: 34.1 kg/m^2^	2	VAT to RCP(≥76%HRmax）	AT	40	24	Exe: 19.8 mL/kg/min CON: 22.1 mL/kg/min	Exercise method: Treadmill Protocol: Ramp-incremental (speed & grade increased every min to exhaustion)
Cuthbertson et al. 2016 (23) UK	Sedentary Adults with MASLD *N* = 50 M/F: 39/11	Age CON: 52.0 yrs Exe: 50.0 yrs BMI CON:29.7 kg/m^2^ Exe: 30.7 kg/m^2^	3–5	30–60%HRR	AT	30–45	16	Exe: 23.7 mL/kg/min CON: 23.2 mL/kg/min	Exercise method: Cycle ergometer Protocol: Step-incremental (start 35 W; +35 W every 3 min to exhaustion)
Keating et al. 2017 (26) UK	Inactive adults with obesity *N* = 29 M/F: 4/25	Age CON:44.2 yrs Exe:45.4 yrs BMI CON:30.8 kg/m^2^ Exe:32.2 kg/m^2^	3	80–85% 1RM	RT	30–60	8	Exe: 23.6 mL/kg/min CON: 21.5 mL/kg/min	Exercise method: cycle ergometer Protocol: Ramp-incremental test (initial power: 35 W for women, 65 W for men; +25 W every 150 s until volitional exhaustion); VO₂peak calculated from the highest 15-s average in the final minute
Abdelbasset et al. 2019 (22) Saudi Arabia	Diabetic Obesity with MASLD *N* = 32 M/F: 19/13	Age CON: 55.2 yrs Exe: 54.4 yrs BMI CON:35.9 kg/m^2^ Exe: 36.3 kg/m^2^	3	80–85%/50% VO_2_peak	HIIT	18	8	Exe: 19.6 mL/kg/min CON: 20.2 mL/kg/min	NR
Stine et al. 2022 (31)	Sedentary Men and women with NASH *N* = 28 M/F: 11/17	Age CON: 45.0 yrs Exe: 52.9yrs BMI CON:35.1 kg/m^2^ Exe: 34.3 kg/m^2^	5	45–55% VO_2_peak	AT	30	20	Exe: 20.3 mL/kg/min CON: 23.9 mL/kg/min-0	Exercise method: Treadmill Protocol: Bruce protocol
Keating et al. 2023 (17) UK	Adults with NASH *N* = 14 M/F: 9/5	Age CON: 61.0 yrs Exe: 53.0yrs BMI CON:38.3 kg/m^2^ Exe: 39.6 kg/m^2^	3	85–95%/60% HRmax	HIIT	7–28	12	Exe: 19.4 mL/kg/min CON: 17.2 mL/kg/min	Exercise method: treadmill or cycle ergometer Treadmill protocol: start at 4 km/h-0% grade for 2 min → 4 km/h-4% grade for 2 min, then increase speed by 1 km/h every 3 min and grade by 1% every 1 min until volitional fatigue or termination criteria. Cycle protocol: after 4-min warm-up at 60 rev/min (RPE 10–12), work-rate is increased by 25 W each minute to exhaustion
Mucinski et al. 2024 (15) USA	Adults with NASH *N* = 24 M/F: 9/15	Age CON: 47.0yrs Exe: 48.7 yrs BMI CON:33.4 kg/m^2^ Exe: 37.5 kg/m^2^	3	90–95%/50% HRmax	HIIT	28	40	Exe: 2.3 L/min CON: 2.1 L/min	Exercise method: Treadmill Protocol: Modified Bruce exercise tolerance test
Willis et al. 2024 (33) UK	Sedentary with MASLD *N* = 24 M/F: 19/5	Age CON: 63.0 yrs Exe: 61.0 yrs BMI CON:31.9 kg/m^2^ Exe: 34.1 kg/m^2^	4	70–75% HRmax	AT	35–50	6	Exe: 28.7 mL/kg/min CON: 27.4 mL/kg/min	Exercise method: Treadmill Protocol: 3 min at 3.5 km/h and 2 min at 5.3 km/h, following a 1% gradient increasing each minute to exhaustion.

### Risk of bias

The methodological quality of the 13 included RCTs was evaluated using the Cochrane RoB 2.0 tool (Supplementary Figure S1). Overall, 2 studies were categorized as having a low risk of bias, while 11 studies were rated as having “some concerns”, and no studies were identified as high risk. The primary sources of concern were deviations from intended interventions and missing outcome data. Notably, blinding of participants and personnel was inherently unfeasible due to the nature of supervised exercise interventions. While these studies were judged as having “some concerns” regarding deviations from intended interventions, this does not necessarily imply a high risk of bias.

### Main results

#### Meta-analysis

**Figure 2:**
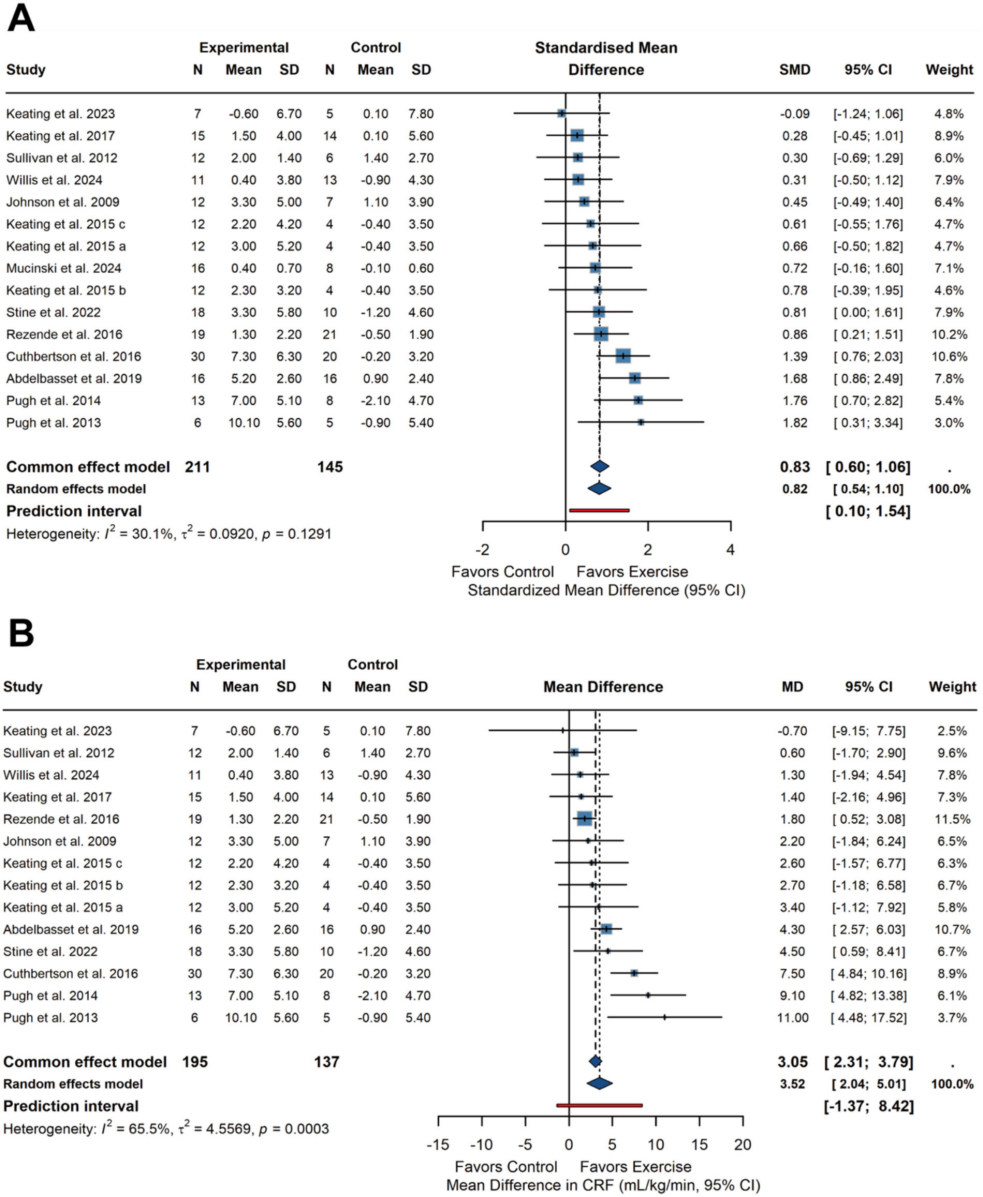
Forest plots of the effect of exercise on cardiorespiratory fitness. **(A)** Pooled standardized mean difference including all eligible comparisons. **(B)**Pooled weighted mean difference in mL/kg/min after excluding Mucinski et al. (2024), which reported CRF in L/min. SMD, standardized mean difference; WMD, weighted mean difference; CRF, cardiorespiratory fitness.

The random-effects meta-analysis demonstrated that exercise interventions significantly improved CRF in individuals with MASLD (Figure 2A), with a pooled SMD of 0.82 (95% CI: 0.54 to 1.10; I^2^ = 30.1%). To improve clinical interpretability, we additionally performed a WMD analysis restricted to studies reporting CRF in mL/kg/min. After excluding Mucinski et al. (2024), which reported CRF in L/min, exercise intervention increased CRF by 3.52 mL/kg/min compared with control conditions (95% CI: 2.04 to 5.01; I^2^ = 65.5%; Figure 2B).

#### Subgroup analysis

Subgroup analyses were conducted to examine whether the effects of exercise on CRF differed according to key intervention and participant characteristics, including exercise type, intervention duration, weekly frequency, session duration, exercise intensity, body weight status, weekly exercise volume, and total cumulative exercise volume (Supplementary Table S2). Overall, no statistically significant between-subgroup differences were observed across these factors.

In the subgroup analysis by exercise type, aerobic training was associated with a significant improvement in CRF (SMD = 0.87, 95% CI: 0.57–1.17; I^2^ = 10.4%), whereas HIIT showed a positive but statistically non-significant effect estimate (SMD = 0.83, 95% CI: −0.15 to 1.81; I^2^ = 68.9%). The single resistance-training comparison also showed a positive but statistically non-significant effect estimate (SMD = 0.28, 95% CI: −0.45 to 1.01). The test for subgroup differences by exercise type was not statistically significant (*χ*^2^ = 2.12, df = 2, *P* = 0.3464). At the comparison level, statistically significant improvements in CRF were identified in 4 of 11 aerobic-training comparisons and 1 of 3 HIIT comparisons, while the single resistance-training comparison was positive but non-significant.

Other subgroup analyses showed broadly consistent beneficial effects of exercise across intervention duration, weekly frequency, session duration, exercise intensity, body weight status, weekly exercise volume, and total cumulative exercise volume. Although some subgroups showed numerically higher point estimates, formal tests for subgroup differences were not statistically significant; therefore, these apparent differences should be interpreted descriptively and cautiously.

#### Dose-response analysis

**Figure 3:**
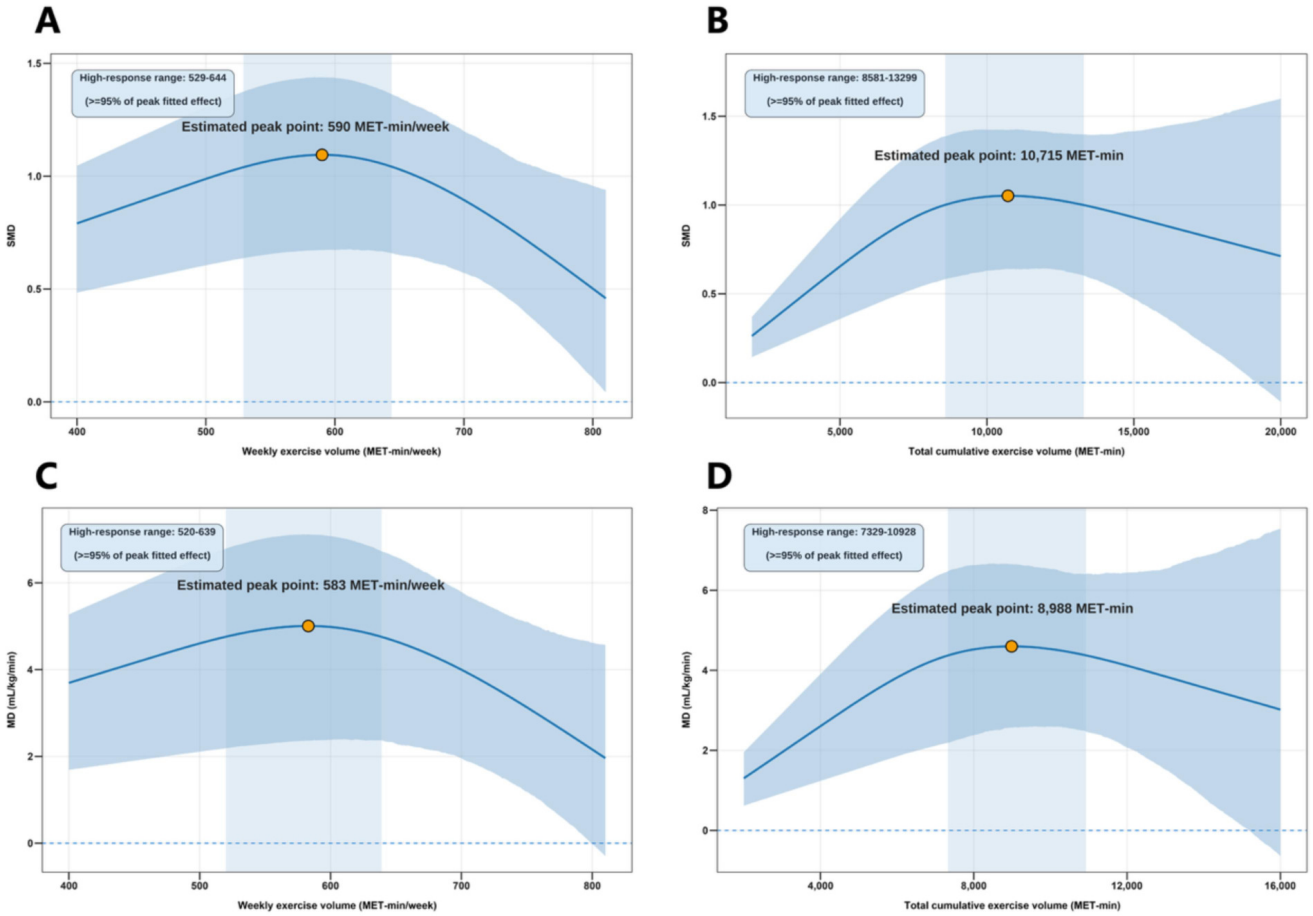
Dose-response relationships between exercise volume and cardiorespiratory fitness. **(A)**, weekly exercise volume using SMD. **(B)**, total cumulative exercise volume using SMD. **(C)**, weekly exercise volume using MD after excluding Mucinski et al. (2024). **(D)**, total cumulative exercise volume using MD after excluding Mucinski et al. (2024). SMD, standardized mean difference; MD, mean difference; CRF, cardiorespiratory fitness.

To characterize the potential non-linear relationship between exercise volume and CRF improvement, we modeled the data using a restricted cubic spline (RCS) function. The dose-response analysis of weekly exercise volume suggested a non-linear pattern, with estimated CRF gains increasing up to approximately 590 MET-min/week before reaching an apparent plateau (Figure 3A). This estimate should be interpreted as an exploratory model-based indicator rather than a definitive optimal dose.

For total cumulative exercise volume, the RCS model suggested that estimated CRF improvement peaked at approximately 10,715 MET-min and attenuated at higher cumulative volumes (Figure 3B). Because fewer studies contributed data at the high-volume end of the curve, this pattern should be interpreted cautiously. In the MD-based sensitivity analysis, after excluding Mucinski et al. (2024), the weekly- and cumulative-volume curves showed broadly consistent patterns, with estimated peak points of approximately 583 MET-min/week and 8,988 MET-min, respectively (Figures 3C, D).

#### Heterogeneity and sensitivity analyses

Sensitivity analyses supported the robustness of the primary findings. In the leave-one-out analysis, the pooled effect remained statistically significant regardless of which individual comparison was removed, with SMDs ranging from 0.75 to 0.87 and I^2^ values ranging from 16.7% to 35.1% (Supplementary Figure S3). After excluding Johnson et al. (2009), which used extrapolated CRF values derived from a structured exercise testing protocol, the pooled effect remained significant and materially unchanged (SMD = 0.84, 95% CI: 0.55 to 1.14; I^2^ = 32.9%). Similarly, after excluding Keating et al. (2017), the only resistance-training study, the pooled effect remained significant (SMD = 0.87, 95% CI: 0.59 to 1.16; I^2^ = 26.3%), indicating that the overall beneficial effect of exercise on CRF was not driven by the inclusion of the resistance-training comparison.In the dose-response sensitivity analysis excluding Keating et al. (2017), the estimated peak points were approximately 587 MET-min/week for weekly exercise volume and 10,534 MET-min for total cumulative exercise volume, showing a broadly similar pattern to the primary SMD-based dose-response analysis (Supplementary Figure S4).

The alternative handling of the multi-arm trial by Keating et al. (2015), in which the three exercise arms were combined and compared with the full control group, also produced results consistent with the primary analysis (SMD = 0.83, 95% CI: 0.53–1.12; I^2^ = 39.8%). The MD-based analysis, performed after excluding Mucinski et al. (2024) because CRF was reported in L/min rather than mL/kg/min, showed a significant improvement in CRF (MD = 3.52 mL/kg/min, 95% CI: 2.04–5.01; I^2^ = 65.5%).

Publication bias was assessed visually using funnel plots and statistically using Begg's rank correlation test. The visual inspection of the funnel plot for Cardiorespiratory Fitness (CRF) revealed a relatively symmetrical distribution of studies. Consistently, Begg's test indicated no significant evidence of potential publication bias (*p* = 0.601). To further verify the robustness of the results, Duval and Tweedie's trim-and-fill method was performed. The analysis estimated zero missing studies, leaving the pooled effect size unchanged (Supplementary Figure S2). These findings suggest that the results regarding the effect of exercise intervention on CRF are robust and not driven by publication bias.

## Discussion

This systematic review and meta-analysis showed that exercise interventions significantly improved CRF in individuals with MASLD. The pooled effect was moderate when expressed as SMD, and the MD-based analysis suggested an average improvement of approximately 3.52 mL/kg/min after excluding the study that reported CRF in L/min. Sensitivity analyses, including leave-one-out analyses, exclusion of the extrapolated CRF study, exclusion of the only resistance-training study, and alternative handling of the multi-arm trial, supported the robustness of the primary findings. Exploratory dose-response analyses suggested a non-linear relationship between exercise volume and CRF improvement, with estimated peak points around 590 MET-min/week and 10,715 MET-min for weekly and total cumulative exercise volume, respectively.

In terms of absolute clinical benefit, the WMD analysis suggested that supervised exercise increased CRF by approximately 3.52 mL/kg/min after excluding Mucinski et al. (2024) (7), which reported CRF in L/min. This magnitude of improvement is approximately equivalent to one metabolic equivalent and may be clinically meaningful, given the established association between higher CRF and lower cardiometabolic risk (16). Nevertheless, this estimate should be interpreted alongside the moderate heterogeneity observed in the WMD analysis and the exclusion of Mucinski et al. (2024).

The exploratory dose-response analysis suggested that CRF improvements increased with exercise volume up to an estimated peak point, followed by an apparent plateau or attenuation at higher volumes. The estimated weekly peak point of approximately 590 MET-min/week falls within the range of current physical activity and MASLD-related exercise recommendations, which generally recommend 150–300 min/week of moderate-intensity physical activity. This finding suggests that CRF gains in individuals with MASLD may require exercise volumes beyond the lower bound of general recommendations (13, 17). For total cumulative exercise volume, the fitted curve suggested that CRF improvement peaked at approximately 10,715 MET-min and attenuated at higher cumulative volumes. This pattern may reflect the balance between sufficient training stimulus and diminishing marginal adaptations at higher exercise volumes (18). However, these estimates should be interpreted cautiously because the number of included trials was limited, dose values were derived from reported intervention characteristics, and fewer studies contributed information at the high-volume end of the curve. Therefore, the estimated peak points should be viewed as exploratory model-based indicators rather than definitive optimal exercise prescriptions.

Subgroup analyses suggested that aerobic exercise significantly improved CRF, whereas HIIT and resistance training showed positive but statistically non-significant effect estimates. This finding appears inconsistent with the broader evidence suggesting that HIIT is often comparable or superior to moderate-intensity continuous training for improving cardiometabolic health (19). However, the absence of a statistically significant subgroup difference by exercise type should not be interpreted as evidence of equivalent effects across exercise modalities. The modality-specific analyses were limited by the small number of HIIT comparisons and the availability of only one resistance-training comparison, which reduced the precision of subgroup estimates and limited the statistical power to detect potential between-modality differences. Therefore, the present findings support an overall beneficial effect of exercise on CRF but remain insufficient to determine the comparative effectiveness of aerobic training, HIIT, and resistance training in MASLD. Given that HIIT has shown comparable or superior efficacy in reducing hepatic steatosis (20, 21), future high-quality RCTs are warranted to clarify its impact on CRF in the MASLD population. Additionally, because only one resistance-training trial was available, the effect of resistance training alone on CRF in MASLD remains uncertain. However, sensitivity analysis excluding this study yielded consistent results, supporting the robustness of the overall finding.

Regarding the interpretation of effect sizes, we observed moderate-to-substantial heterogeneity in the WMD analysis (I^2^ = 65.5%), whereas the SMD analysis showed lower heterogeneity (I^2^ = 30.1%). This discrepancy is likely attributable to variation in CRF assessment protocols and reporting units across the included studies. Testing modalities included cycle ergometers (22–26), treadmills (27–33), and laboratory-based extrapolated exercise tests (24), creating inherent variability in absolute VO₂peak values. Nevertheless, Singh et al. reported that various CRF assessment methods are all valid predictors of cardiovascular outcomes, providing a theoretical basis for pooling these diverse data (34).The use of SMDs helped standardize these differences and provided a more comparable estimate of the relative intervention effect across diverse testing conditions.

The primary strength of this study lies in its methodological novelty. To our knowledge, this is the first systematic review to apply a restricted cubic spline modeling framework to characterize the potential non-linear dose-response relationship between exercise volume and CRF in individuals with MASLD. Unlike previous meta-analyses that treated exercise dose as a binary or linear variable, our approach provided exploratory model-based estimates of the exercise-volume range associated with greater CRF improvement. These findings may help generate hypotheses for refining exercise prescriptions, although they should not be interpreted as definitive optimal dose thresholds.

However, several limitations must be acknowledged. First, because most included trials were conducted before the adoption of the MASLD nomenclature, participants were originally diagnosed using previous NAFLD/NASH criteria. Although these populations are expected to substantially overlap with the current MASLD framework, some degree of diagnostic heterogeneity may remain. Second, the quantification of exercise volume relied on estimations from the Compendium of Physical Activities rather than direct physiological measurements, such as calorimetry. Although this approach is commonly used in exercise meta-analyses, it may introduce estimation error regarding the exact energy expenditure. In addition, MET-based quantification may be less precise for resistance training than for aerobic exercise because it does not fully capture external load, rest intervals, movement tempo, or neuromuscular effort. However, only one resistance-training study was included, and sensitivity analysis excluding this study yielded consistent results, suggesting that the main findings were not driven by the resistance-training dose estimate. Third, as the majority of included RCTs had intervention durations between 8 and 16 weeks, our findings, particularly the attenuated cumulative dose-response pattern, primarily reflect short-term physiological adaptations. The long-term effects of high-volume exercise interventions lasting more than 6 months on CRF remain to be elucidated. Finally, although the primary analysis showed a robust overall effect, the statistical power for subgroup and dose-response analyses was limited by the small number of eligible studies, particularly for HIIT, resistance training, and higher cumulative exercise volumes.

## Conclusions

Supervised structured exercise improves CRF in individuals with MASLD. Exploratory dose-response analyses suggested non-linear associations between exercise volume and CRF improvement, with estimated peak points around 590 MET-min/week and 10,715 MET-min for weekly and total cumulative exercise volume, respectively. These model-based estimates should not be interpreted as definitive optimal exercise doses. Future long-term trials are needed to confirm these dose-response patterns and refine exercise prescriptions for individuals with MASLD.

## Data Availability

The original contributions presented in the study are included in the article/Supplementary Material, further inquiries can be directed to the corresponding author/s.
