# Supplementary material for: Effects of exercise on cardiorespiratory fitness in individuals with MASLD: a systematic review and dose-response meta-analysis
Source: Front Sports Act Living. 2026 Jun 19;8:1854220. doi: 10.3389/fspor.2026.1854220 (PMC13330729; doi:10.3389/fspor.2026.1854220)
Supplement: Supplementary file 1 [file Datasheet1.docx]

**Supplementary Material:**

**Table S1. Detailed Search Strategies**

**Pubmed**

| Step | Search Terms |
| --- | --- |
| #1 | ("Non-alcoholic Fatty Liver Disease"[Mesh] OR "Non-alcoholic Fatty Liver Disease"[Title/Abstract] OR "Fatty Liver"[Title/Abstract] OR "NAFLD"[Title/Abstract] OR "NASH"[Title/Abstract] OR "non-alcoholic steatohepatitis"[Title/Abstract] OR "nonalcoholic steatohepatitis"[Title/Abstract] OR "metabolic dysfunction-associated steatotic liver disease*"[Title/Abstract] OR "MASLD"[Title/Abstract] OR "metabolic associated fatty liver disease*"[Title/Abstract] OR "MAFLD"[Title/Abstract] OR "steatohepatitis"[Title/Abstract] OR "steatosis"[Title/Abstract] OR "MASH"[Title/Abstract]) |
| #2 | ("Exercise"[Mesh] OR "Exercise Therapy"[Mesh] OR "Exercise"[Title/Abstract] OR "Training"[Title/Abstract] OR "Intervention"[Title/Abstract] OR "Endurance"[Title/Abstract] OR "Strength"[Title/Abstract] OR "Concurrent"[Title/Abstract] OR "combined"[Title/Abstract] OR "Aerobic"[Title/Abstract] OR "Resistance"[Title/Abstract] OR "Interval"[Title/Abstract] OR "HIIT"[Title/Abstract]) |
| #3 | ("Randomized Controlled Trial"[Mesh] OR "Randomized Controlled Trial"[Title/Abstract] OR "randomized"[Title/Abstract] OR "placebo"[Title/Abstract] OR "randomly"[Title/Abstract] OR "trial"[Title/Abstract] OR "RCT"[Title/Abstract]) |
| #4 | #1 AND #2 AND #3 |

**Embase**

| Steps | Search Terms |
| --- | --- |
| #1 | 'nonalcoholic fatty liver'/exp OR 'steatohepatitis'/exp OR 'liver steatosis'/exp OR 'metabolic dysfunction-associated steatotic liver disease':ti,ab OR 'masld':ti,ab OR 'mafld':ti,ab OR 'nafld':ti,ab OR 'nash':ti,ab OR 'fatty liver':ti,ab |
| #2 | 'exercise'/exp OR 'exercise therapy'/exp OR 'training'/exp OR 'aerobic exercise'/exp OR 'resistance training'/exp OR 'exercise':ti,ab OR 'training':ti,ab OR 'intervention':ti,ab OR 'hiit':ti,ab OR 'aerobic':ti,ab OR 'resistance':ti,ab |
| #3 | 'randomized controlled trial'/exp OR 'randomization'/exp OR 'randomized controlled trial':ti,ab OR 'random*':ti,ab OR 'rct':ti,ab OR 'trial':ti,ab |
| #5 | #1 AND #2 AND #3 |

**Web of science**

| Steps | Search Terms |
| --- | --- |
| #1 | TS = ("metabolic dysfunction-associated steatotic liver disease*" OR "MASLD" OR "MAFLD" OR "NAFLD" OR "NASH" OR "non-alcoholic fatty liver" OR "steatohepatitis" OR "steatosis" OR "fatty liver") |
| #2 | TS = ("exercise*" OR "training" OR "intervention*" OR "aerobic" OR "resistance" OR "HIIT" OR "strength" OR "endurance" OR "concurrent") |
| #3 | TS = ("randomized controlled trial" OR "randomized" OR "randomly" OR "placebo" OR "RCT" OR "trial") |
| #4 | #1 AND #2 AND #3 |

**Table S2: Subgroup analyses of the effects of exercise on CRF**

| Category | Subgroup | k | SMD | 95%CI | I^2^ | P_between |
| --- | --- | --- | --- | --- | --- | --- |
| type | AT | 11 | 0.87 | 0.57, 1.17 | 10.4 | 0.346 |
|  | HIIT | 3 | 0.83 | -0.15, 1.81 | 68.9 | 0.346 |
|  | RT | 1 | 0.28 | -0.45, 1.01 |  | 0.346 |
| time | <40 | 8 | 0.94 | 0.51, 1.36 | 32.3 | 0.437 |
|  | ≥40 | 7 | 0.71 | 0.33, 1.09 | 29.3 | 0.437 |
| frequency | ≤3 | 9 | 0.71 | 0.37, 1.05 | 11.7 | 0.356 |
|  | >3 | 6 | 1 | 0.48, 1.51 | 47.9 | 0.356 |
| duration | ≤12 | 8 | 0.6 | 0.19, 1.01 | 25.8 | 0.112 |
|  | >12 | 7 | 1.03 | 0.70, 1.36 | 17.8 | 0.112 |
| intensity | ≥6 | 6 | 0.73 | 0.27, 1.20 | 42.3 | 0.631 |
|  | 3-5.9 | 9 | 0.88 | 0.50, 1.25 | 27.3 | 0.631 |
| Body weight | ≥35 | 5 | 0.72 | 0.10, 1.33 | 48.5 | 0.708 |
|  | 30-35 | 10 | 0.85 | 0.52, 1.17 | 26.3 | 0.708 |
| weekly_volume | high weekly volume | 8 | 0.94 | 0.53, 1.35 | 29.8 | 0.407 |
|  | low weekly volume | 7 | 0.7 | 0.29, 1.10 | 33.6 | 0.407 |
| total_volume | low total volume | 8 | 0.6 | 0.19, 1.01 | 25.8 | 0.112 |
|  | high total volume | 7 | 1.03 | 0.70, 1.36 | 17.8 | 0.112 |

**Table S3. Sensitivity analyses of the pooled SMD effect**

| Analysis | k | SMD | 95%CI | p_overall | I^2^ | p_heterogeneity |
| --- | --- | --- | --- | --- | --- | --- |
| Excluding Johnson et al. 2009 | 14 | 0.84 | 0.55, 1.14 | < 0.001 | 32.93 | 0.11 |
| Excluding Keating et al. 2017 | 14 | 0.87 | 0.59, 1.16 | < 0.001 | 26.29 | 0.17 |
| Keating 2015 combined arms | 13 | 0.83 | 0.53, 1.12 | < 0.001 | 39.76 | 0.07 |

**Table S4 The diagnostic criteria table**

| Study (Country) | Population | NAFLD/MASLD Diagnostic Criteria |
| --- | --- | --- |
| Johnson et al. 2009 (Australia) | Obese subjects | Obese (BMI >=30 kg/m2), sedentary, low alcohol intake (0-20 g/day). Excluded: acute/chronic disease (other than obesity/hypertension), lipid-lowering medications, fasting glucose >=7.0 mmol/L. Hepatic triglyceride content (HTGC) measured by 1H-MRS. |
| Sullivan et al. 2012 (USA) | Obese subjects with NAFLD | IHTG content >10% by MRS. Excluded: chronic liver disease other than NAFLD, alcohol misuse (Michigan Alcohol Screening Test score >4), diabetes, plasma TG >400 mg/dL. Sedentary (<1 h/week exercise), weight stable. |
| Pugh et al. 2013 (UK) | Adults with NAFLD | Diagnosis based on chronically elevated ALT (>=41 U/L for >=6 months) with echobright liver on abdominal ultrasound. Confirmed by IHCTG >=5.56% on 1H-MRS. Excluded: viral hepatitis, autoimmune hepatitis, primary biliary cirrhosis, other metabolic liver diseases, type 2 diabetes, excessive alcohol intake. Non-smokers, sedentary. |
| Pugh et al. 2014 (UK) | Obese adults with NAFLD | NAFLD defined as liver fat >=5.5% by 1H-MRS with elevated transaminases. Excluded: viral hepatitis, autoimmune hepatitis, PBC, metabolic disorders, excessive alcohol intake, type 2 diabetes. Obese (waist circumference >=94 cm men, >=80 cm women), sedentary, non-smokers. |
| Keating et al. 2015 (Australia) | Overweight/obese adults | Inactive (<3 days/week structured exercise), BMI >25 kg/m2. Excluded: lipid-lowering or insulin-sensitizing medication, alcohol >20 g/day, secondary causes of steatohepatitis, alcoholic liver disease, viral hepatitis. Liver fat measured by 1H-MRS. |
| Rezende et al. 2016 (Brazil) | Postmenopausal women with NAFLD | Biopsy-proven NAFLD with histological grading by NAFLD Activity Score (NAS). Excluded: hormone therapy, alcohol >20 g/day, HIV, hepatitis B/C, other chronic liver diseases, physical/cardiovascular limitations. Postmenopausal, non-smokers. |
| Cuthbertson et al. 2016 (UK) | Sedentary adults with NAFLD | NAFLD diagnosed clinically by hepatologist after exclusion of drug causes, viral/autoimmune hepatitis (negative hepatitis B/C serology and auto-antibody screen), PBC, metabolic disorders (alpha1-antitrypsin deficiency, Wilson disease). NAFLD defined as mean IHCL >5.3% by 1H-MRS. Inclusion: sedentary (<2 h/week low-intensity PA), non-smokers, alcohol <14 (F)/<21 (M) units/week. Excluded: T2DM, ischemic heart disease, contraindications to exercise. |
| Keating et al. 2017 (UK) | Inactive obese adults | Inactive (<3 days/week or <150 min/week moderate exercise), BMI >25 kg/m2. Excluded: alcohol >20 g/day, lipid-lowering/insulin-sensitizing agents, recent weight change (>2 kg). Liver fat by 1H-MRS. |
| Abdelbasset et al. 2019 (Saudi Arabia) | Diabetic obese with NAFLD | Diagnostic criteria based on NAFLD guidelines for the Asia-Pacific region. Type 2 diabetes, BMI >=35 kg/m2, age 45-60 years. Hepatic fat content assessed by MRI. |
| Stine et al. 2022 (USA) | Sedentary adults with NASH | Biopsy-confirmed NASH per NASH Clinical Research Network (CRN) histological scoring system. Sedentary (<90 min PA/week). Excluded: HbA1c >9%, other chronic liver diseases, excessive alcohol consumption, inability to exercise. |
| Keating et al. 2023 (UK) | Adults with NASH | Inclusion: age 18-70, not meeting physical activity guidelines (<150 min moderate or <75 min vigorous aerobic exercise/week), NASH confirmed by liver biopsy. Exclusion: significant contraindications to exercise testing/training, or any other reason limiting study participation. |
| Mucinski et al. 2024 (USA) | Adults with MASH | Diagnostic liver biopsy for suspected MASLD; NAS >4/8; BMI 25-50 kg/m2; >=3/5 metabolic syndrome criteria. Sedentary men and women. |
| Willis et al. 2024 (UK) | Men with MASLD | MASLD defined per updated diagnostic criteria: PDFF >=5.56% by 1H-MRS + >=1 cardiometabolic risk factor, absence of excessive alcohol (<30 g/day) or other secondary aetiologies. Inactive men, age 30-75, overweight/obese. Excluded: type 2 diabetes managed other than lifestyle/metformin. |

**Table S5 MET calculation for Intervention protocol**

| Comparison ID | Protocol extracted | High/continuous MET | High/continuous min per session | Low/recovery MET | Low/recovery + warm/cool min per session | Frequency (sessions/week) | Duration (weeks) | Session MET-min | Weekly dose (MET-min/week) | Total dose(MET-min) |
| --- | --- | --- | --- | --- | --- | --- | --- | --- | --- | --- |
| Johnson 2009 | 50–70% VO₂peak; 30–45 min/session; 3 sessions/week; 4 weeks | 5.8 | 37.5 | 0.0 | 0.0 | 3.0 | 4.0 | 217.5 | 652.5 | 2610.0 |
| Sullivan 2012 | 45–55% VO₂peak/HRR; 30–60 min/session; 5 sessions/week; 16 weeks; reported average 224 min/week | 3.5 | 45.0 | 0.0 | 0.0 | 5.0 | 16.0 | 157.5 | 787.5 | 12600.0 |
| Pugh 2013 | 30–60% HRR; 30–45 min/session; 4 sessions/week; 16 weeks | 4.5 | 37.5 | 0.0 | 0.0 | 4.0 | 16.0 | 168.8 | 675.0 | 10800.0 |
| Pugh 2014 | 30–60% HRR; 30–45 min/session; 4 sessions/week; 16 weeks | 4.5 | 37.5 | 0.0 | 0.0 | 4.0 | 16.0 | 168.8 | 675.0 | 10800.0 |
| Keating 2015 HI:LO | High-intensity low-volume aerobic exercise: 70% VO₂peak; 45 min/session; 3 sessions/week; 8 weeks | 5.8 | 45.0 | 0.0 | 0.0 | 3.0 | 8.0 | 261.0 | 783.0 | 6264.0 |
| Keating 2015 LO:HI | Low-to-moderate intensity high-volume aerobic exercise: 50% VO₂peak; 60 min/session; 4 sessions/week; 8 weeks | 4.0 | 60.0 | 0.0 | 0.0 | 4.0 | 8.0 | 240.0 | 960.0 | 7680.0 |
| Keating 2015 LO:LO | Low-to-moderate intensity low-volume aerobic exercise: 50% VO₂peak; 45 min/session; 3 sessions/week; 8 weeks | 4.0 | 45.0 | 0.0 | 0.0 | 3.0 | 8.0 | 180.0 | 540.0 | 4320.0 |
| Cuthbertson 2016 | Progressed from 3×30 min at 30% HRR to 5×45 min at 60% HRR by week 12; 16 weeks total | 4.0 | 37.5 | 0.0 | 0.0 | 4.0 | 16.0 | 150.0 | 600.0 | 9600.0 |
| Rezende 2016 | Progressed from 30-50min, intensty from VAT up to 10% below RCT, 2 sessions/week, 24 weeks. | 5.8 | 40.0 | 0.0 | 0.0 | 2.0 | 24.0 | 232.0 | 464.0 | 11136.0 |
| Keating 2017 PRT | 10 exercises/session; 8–12 repetitions; 2–3 sets/exercise; 80–85% 1RM; 3 sessions/week; 8 weeks | 5.8 | 45.0 | 0.0 | 0.0 | 3.0 | 8.0 | 261.0 | 783.0 | 6264.0 |
| Abdelbasset 2019 | 5-min warm-up; 3×4-min cycling at 80–85% VO₂max; 2-min recovery at 50% VO₂max between sets; 5-min cool-down; 3 sessions/week; 8 weeks | 7.0 | 12.0 | 3.0 | 14.0 | 3.0 | 8.0 | 126.0 | 378.0 | 3024.0 |
| Stine 2022 | 45–55% VO₂peak; 30 min/session; 5 sessions/week; 20 weeks | 3.5 | 30.0 | 0.0 | 0.0 | 5.0 | 20.0 | 105.0 | 525.0 | 10500.0 |
| Keating 2023 | 5-min warm-up at 60% HRmax; 4×4-min intervals at 85–95% HRmax; 3-min active recoveries at ~60% HRmax; 5-min cool-down; progression weeks 1–4; 3 sessions/week; 12 weeks | 7.5 | 14.0 | 3.0 | 17.5 | 3.0 | 12.0 | 157.5 | 472.5 | 5670.0 |
| Mucinski 2024 | Supervised HIIT; 90–95%/50% HRmax; 4*（4+3） min/session; 3 sessions/week; 40 weeks | 8.0 | 16.0 | 3.0 | 12.0 | 3.0 | 40.0 | 164.0 | 492.0 | 19680.0 |
| Willis 2024 | 70–75% HRmax; 4 sessions/week; session duration progressed from 35 to 50 min; 6 weeks | 4.0 | 42.5 | 0.0 | 0.0 | 4.0 | 6.0 | 170.0 | 680.0 | 4080.0 |

MET values were assigned primarily according to ACSM relative-intensity classifications based on the reported exercise-prescription parameters, including %VO₂peak/%VO₂max, %HRmax, %HRR, RPE, exercise modality, session duration, weekly frequency, and intervention duration. The Compendium of Physical Activities was used as a supplementary reference to identify the closest activity category for each exercise mode. When an exact activity category was not directly applicable, MET values were assigned conservatively according to the ACSM intensity range and the expected absolute metabolic capacity of individuals with obesity or MASLD. For HIIT protocols, high-intensity and recovery intervals were calculated separately when segment-specific information was available. For resistance training, MET-based quantification was considered approximate because it may not fully capture external load, rest intervals, movement tempo, or neuromuscular effort.

**
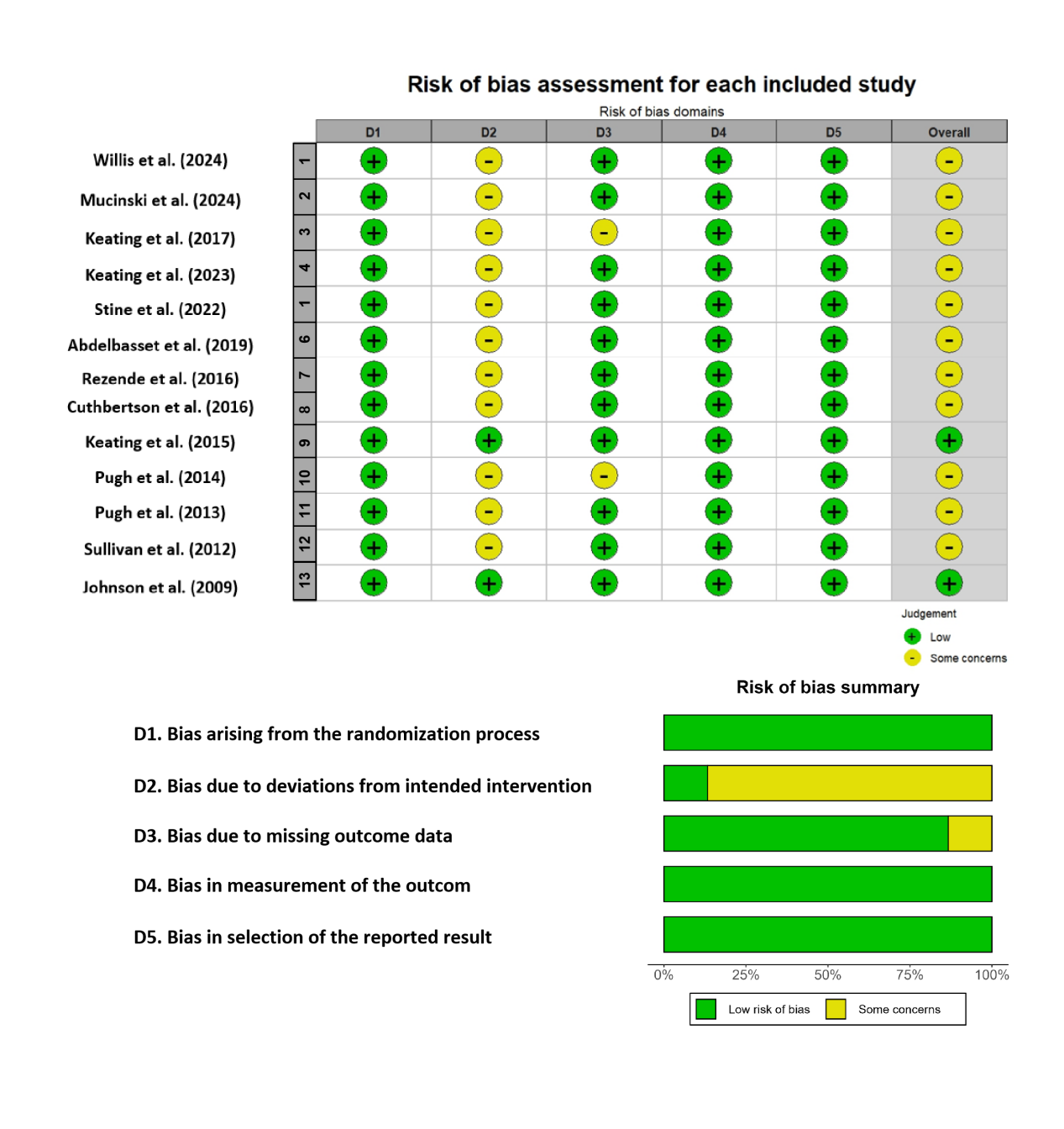
**

**Figure S1 Risk of bias summary**

**
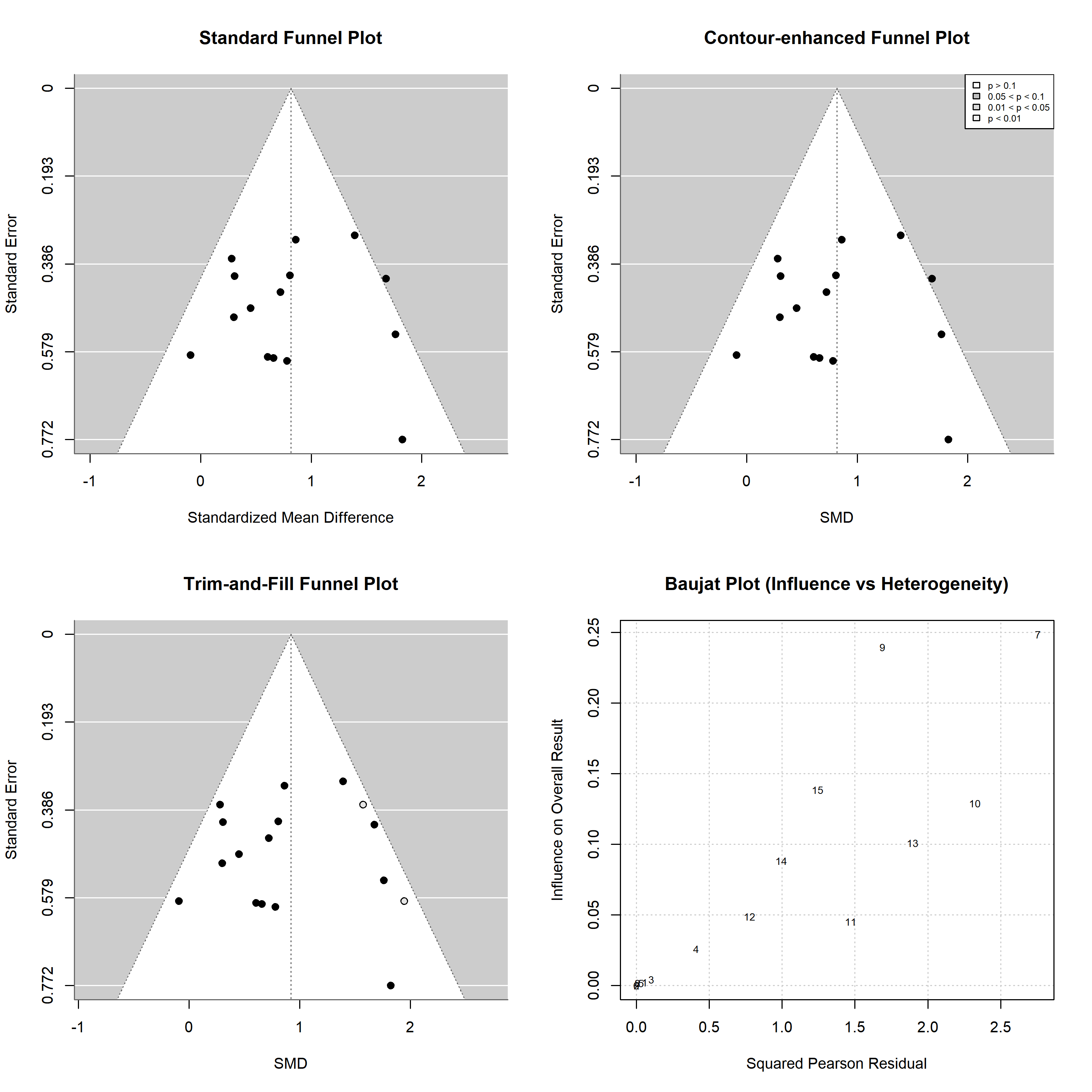
**

**Figure S2: Assessment of publication bias for cardiorespiratory fitness**

Begg’s Test Result: Rank Correlation Test for Funnel Plot Asymmetry; Kendall's tau = -0.0980, p = 0.6009; Trim-and-Fill Result: Estimated missing studies: 0; Estimated number of missing studies on the left side: 0 (SE = 2.6144)


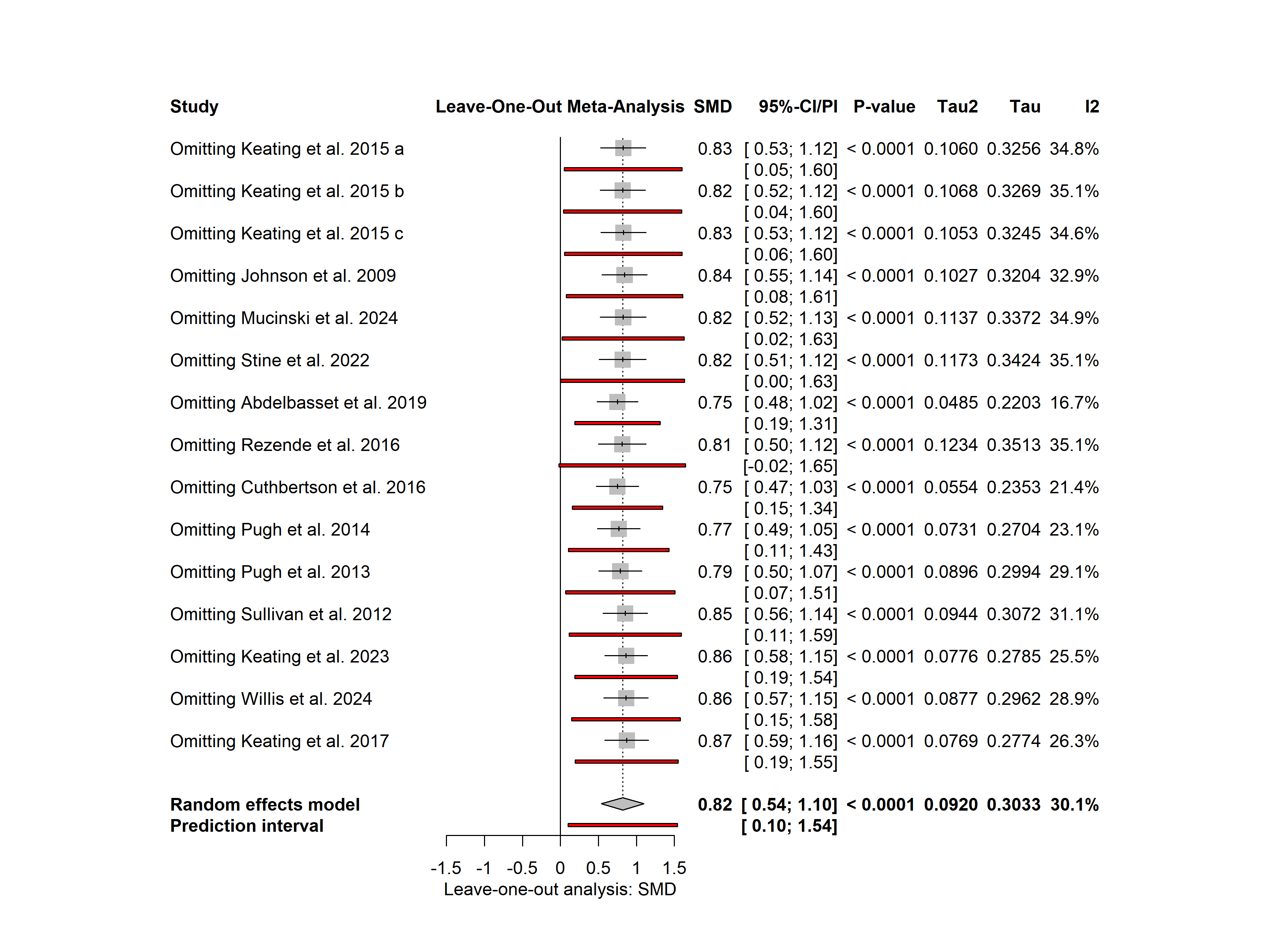
**Figure S3. Leave-one-out sensitivity analysis of the effect of exercise on cardiorespiratory fitness.**The pooled standardized mean difference (SMD) was recalculated after sequentially omitting each individual comparison. The pooled effects remained statistically significant across all iterations, indicating that the overall effect of exercise on cardiorespiratory fitness was not driven by any single comparison. SMD, standardized mean difference; CRF, cardiorespiratory fitness.


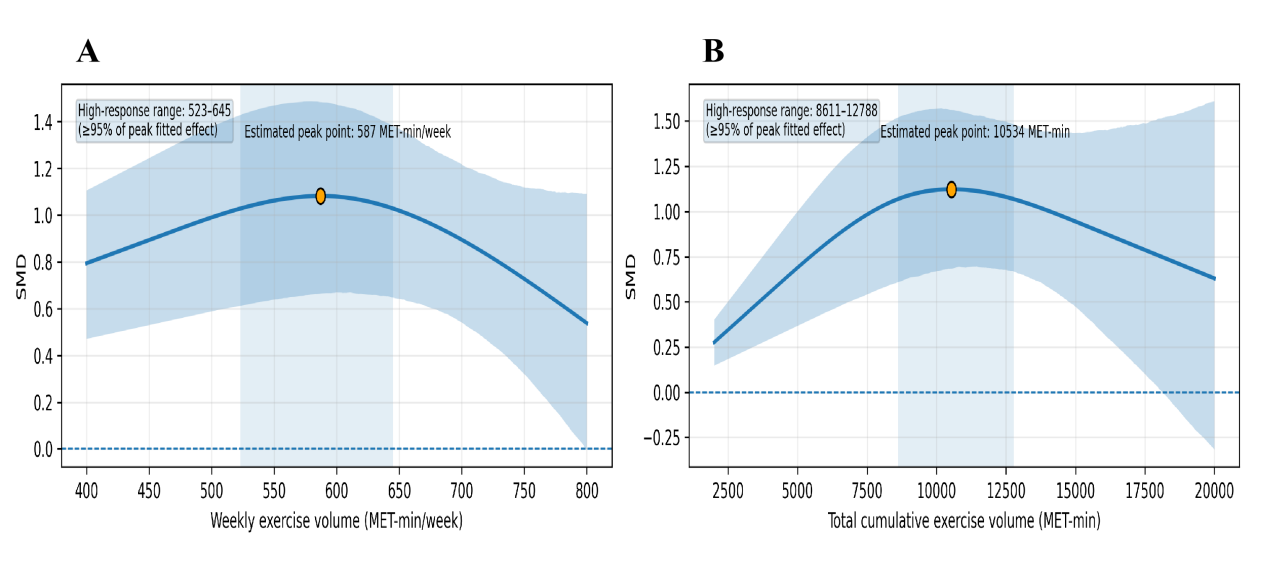


**Figure S4. Dose-response sensitivity analysis excluding the resistance-training study.**The restricted cubic spline dose-response analysis was repeated after excluding Keating et al. (2017), the only resistance-training study. A, weekly exercise volume using SMD. B, total cumulative exercise volume using SMD. The circle indicates the estimated peak point of the fitted curve, and the shaded band indicates the estimated high-response range, defined as the dose range corresponding to at least 95% of the peak fitted effect. These estimates should be interpreted as exploratory model-based indicators rather than definitive optimal exercise doses. SMD, standardized mean difference.
